# Tailoring High-Entropy Oxides as Emerging Radiative Materials for Daytime Passive Cooling

**DOI:** 10.1021/acs.chemmater.3c01205

**Published:** 2023-10-23

**Authors:** Costanza Borghesi, Claudia Fabiani, Roberto Bondi, Loredana Latterini, Ivano E. Castelli, Anna Laura Pisello, Giacomo Giorgi

**Affiliations:** † Department of Civil & Environmental Engineering (DICA), University of Perugia, Via G. Duranti 93, Perugia 06125, Italy; ‡ CIRIAF − Interuniversity Research Centre, University of Perugia, Perugia 06125, Italy; ¶ Department of Engineering, Università degli Studi di Perugia, Via G. Duranti 93, Perugia 06125, Italy; § CIRIAF − Interuniversity Research Centre, University of Perugia, Via G. Duranti 67, Perugia 06125, Italy; ∥ Nano4Light Lab, Department of Chemistry, Biology and Biotechnology, University of Perugia, Via Elce di sotto 8, Perugia 06123, Italy; ⊥ Department of Energy Conversion and Storage, Technical University of Denmark, DK-2800 Kgs. Lyngby, Denmark; # CNR-SCITEC, Via Elce di sotto 8, Perugia, 06123, Italy

## Abstract

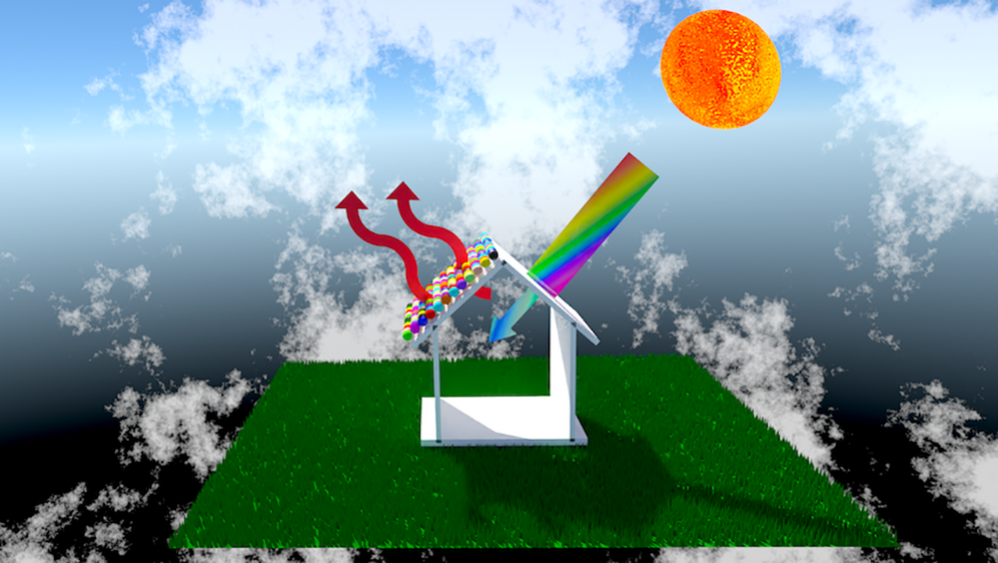

In the framework of intense research about high-entropy materials and their applications in energy-oriented technologies, in the present work, we discuss the potential applicability of selected oxides and of the alloys they form at different concentrations for daytime radiative cooling implementation. In particular, by combining density functional theory and the finite difference method, we provide an unbiased, scattering-free description of structural, electronic, and dynamic features of the best candidates, showing the required strong radiative properties for passive cooling while offering the benefits of affordability and compatibility with commercial coating fabrication processes.

## Introduction

The rise of the CO_2_ concentration in the atmosphere represents an extremely severe and harmful issue intertwined with the global temperature increase. This concentration is rising primarily because of fossil fuel consumption for energy production. Heating, ventilation, and air conditioning systems (HVAC) play a primary role in this uncomfortable scenario, highly impacting the anthropogenic CO_2_ emissions, exploiting electricity mainly produced from nonrenewable fossil fuels, and, in many cases, responsible for local heat dissipation as well. Most air conditioning (AC) systems still work with those technologies that release waste heat into the environment with subsequent outdoor local temperature increases.^1^ Some of these outdated technologies still use hydrofluorocarbons, despite their largely demonstrated hazardousness.^2^ The tight connection between the climate crisis and artificial heating/cooling is thus clear and threatens to become more severe as the global population grows. In particular, urban contexts, where currently 68% of the global population lives, are characterized by local overheating—typically referred to as Urban Heat Islands (UHIs)—with even more impact, as documented in more than 300 cities around the globe.^3−7^ Hence, the investigation of renewable energy sources has been (and still is) a topic of deep interest, as demonstrated by the many pieces of research focusing on solar energy conversion processes, a consequence of recently discovered high performing materials (see, among the others, refs (8−12)). However, as the energy–environment nexus is getting more and more evident, the time has come to combine energy production from renewables with strategies to mitigate detrimental urban overheating effects.^13^

In this direction, an extremely powerful approach has been proposed in the past decade to capitalize on a physical phenomenon, i.e., passive radiative cooling,^14−21^ by which an object is able to dissipate heat at specific frequencies. Passive radiative cooling is a natural nocturnal phenomenon (in a highly thermally emissive regime) that can cool surfaces without energy consumption. Passive coolers directly emit heat through a transparent spectral window of the atmosphere (the so-called “atmospheric sky window”, 8–13 μm) into the cold universe, which, with a temperature of 3 K, functions as an infinite heat sink. Therefore, when applied over buildings’ envelopes, they have been demonstrated to reach subambient surface temperature, improving indoor–outdoor comfort and saving energy in the built environment.

Because of its impressive potential and inherent low costs, such a technique attracts deep interest from the paint production and building construction industries. It has been shown indeed that radiative cooling also occurs during the day^16,22,23^ and can be used to cool a surface below ambient temperature even under direct sunlight if the thermal emission of the surface through the sky window exceeds its absorption of sunlight. Nevertheless, the transition from nocturnal to diurnal conditions drastically changes the scenario, and exploiting radiative cooling turns out to be a challenging task. Accordingly, devices able to induce passive daytime radiative cooling (PDRC) are under intense investigation. These devices are formed by a reflective layer (usually a metallic element) and a radiative cooling layer^17,20^ where the latter is in charge of minimizing the absorption in the visible (VIS) region and of emitting in specific wavebands in the mid-infrared (IR).^16,22,24^ The performance of the cooler deteriorates as fractions of the incident solar light are absorbed. Thus, the first *mandatory* feature is a material that minimizes the solar absorption: absorbing 10% of the solar radiation would indeed correspond to a rise of *T* from ∼200 K to room *T*, frustrating the final goal of such a setup.^16^ The intrinsic difficulty in finding a single material able to properly deal with the IR and VIS region has motivated the usage of metamaterials and expensive (nano)photonic structures.^16,17,22^ Initial attempts to find a single material with interesting properties have focused on CaCO_3_^25^ and other Mg-based compounds^26^ and on porous materials.^27^

The evident lack of atomistic knowledge of the structural and optoelectronic features of the materials constituting the radiative cooling layer is boosting research in this direction. Only very recently have a few papers focused on the metrics (i.e., a suitable bandgap larger than the upper bound of energy in the solar spectrum, 4.13 eV, and a high number of IR-active optical resonance phonon modes in the sky window) accessible from first-principles calculations. These contributions compared the performance of SiO_2_ (the standard material for PDRC) and BaSO_4_ (a suggested alternative), paving the way toward the usage of ab initio atomistic simulations for innovative material development.^28^

Our aim is here to theoretically predict novel materials by exploiting the concepts behind the formation of high-entropy materials^29,30^ and specifically of high-entropy oxides (HEOs).^31^ The concept that encompasses the existence of such materials is that, by increasing the number of ionic components,^32^ the entropic factor (Δ*S*_mix_) becomes dominant in the Gibbs free energy equation. As a consequence of their broad structural diversity, this class of materials has been recently shown to have a huge potential in several device-oriented applications, among others, in dielectric uses,^33^ in ion batteries,^34,35^ in photo-^36^ and electrocatalysis,^37,38^ in energy storage,^39^ and in low thermal conductivity.^40^ Therefore, in order to perform our analysis, we initially start with the fluorite polymorph of Y_2_Ce_2_O_7_,^41^ an oxide composed of Y_2_O_3_^42^ and CeO_2_, which has been recently reported to have very appealing features as a material for PDRC—both free-standing and Fe-doped—embodying the characteristics of a good solar reflector and of an efficient IR emitter.^43^ Keeping in mind the suggested metrics for an ideal radiator (vide supra),^28^ we apply them to such precursor compounds that we in parallel have synthesized and initially characterized, to the intermediates (still synthesized and partly characterized), and to the final HEO (Y_0.25_Sc_0.25_Ga_0.25_In_0.25_)_2_Si_2_O_7_ (hereafter also (YSGI)_2_Si_2_O_7_) resulting from our theoretical screening study. Our analysis reveals that (YSGI)_2_Si_2_O_7_ has superior features as a material for PDRC: it indeed embodies the features of a wide bandgap and (almost) infinite dispersion material (frustrating any residual possible excitation) and those of an excellent IR emitter (smoothly and evenly spreading IR-active modes in the atmospheric sky window).

Thus, combining experimental and theoretical efforts, our present analysis aims to pave the way to the search for environmentally friendly and sustainable materials for passive daytime radiative cooling that can possibly gain wide commercial application with a particular focus on the built environment, with the final aim of mitigating urban heat islands. Importantly, to the best of our knowledge, our work discloses for the first time a new application, as materials for PDRC, for high-entropy oxides, further broadening the range of applications of this class of materials that promise to be the philosopher’s stone of materials science in the years to come.

## Methods

First-principles calculations were performed by means of the Vienna Ab-initio Simulation Package (VASP)^44−47^ using the electron exchange–correlation functional of Perdew–Burke–Ernzerhof (PBE)^48^ for structural optimization. A cutoff energy of 520 eV is used in the projector augmented wave (PAW) method.^49,50^ A 2 × 2 × 2 and a 3 × 2 × 5 k-point mesh was exploited for sampling the Brillouin zone (BZ) of the cubic and of the monoclinic system, respectively. Phonons were computed using the PHONOPY package with the finite displacement method,^51^ employing a 2 × 1 × 2 supercell for the monoclinic compounds to capture the dynamics of the oxides accurately (88 atoms). For the phonon calculation, 2 × 2 × 2 (cubic) and 2 × 3 × 3 (monoclinic) k-point meshes were applied to the systems. All atoms of the supercell have been fully optimized (ions and lattice parameters) until forces were lower than 0.005 eV/Å. To improve over the PBE calculated ones, the electronic properties have been calculated by means of the Heyd–Scuseria–Ernzerhof (HSE06) hybrid functional.^52^ The quasi-particle (QP) energies for (YSGI)_2_Si_2_O_7_ are calculated within the “one-shot” *G*_0_*W*_0_ framework, adding 1078 empty states above the valence band maximum (VBM), which correspond to ∼140 eV, and sampling the BZ with 16 k-points. The number of frequency points is set to 120. The plane-wave cutoff is the default one (400 eV), while the cutoff for response function is set to 267 eV. The optical excitation energies and spectra are obtained solving the Bethe–Salpeter equation (BSE) in order to include excitonic and local-field effects.^53^ The Tamm–Damcoff approximation is here considered,^53,54^ while 20 (20) occupied (unoccupied) states are used to build up the excitonic matrix.

In order to obtain random solid solutions with controlled compositions on each sublattice and to build all structural input files of the generated Special Quasirandom Structures (SQS)^55^ for the considered alloys we exploited the mcsqs^56^ Monte Carlo SQS code of the Alloy Theoretic Automated Toolkit (ATAT).^57−59^ The mcsqs algorithm exploits the cluster expansion theory to find the minimally sized supercell approximation whose cluster correlation functions as the best possible match to those of the targeted random structure. Using the output of the VASP data, we have processed the resultant ComputedEntry (extrated from Materials Project API)^60^ using the MaterialsProjectCompatibility class of pymatgen’s compatibility module to apply adjustments to the energies of the oxide anions to calculate the convex hull and eventually generate the phase diagram.

## Results and Discussion

**Figure 1:**
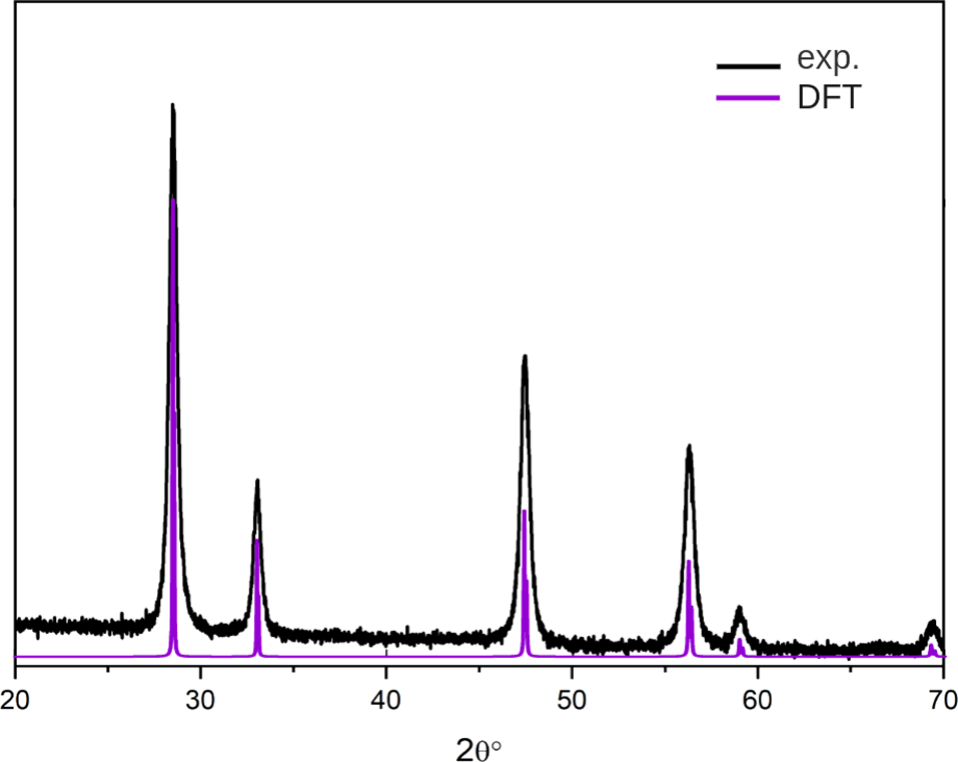
Experimental (black) and theoretical (violet) X-ray powder diffraction pattern of cubic Y_2_Ce_2_O_7_.

**Figure 2:**
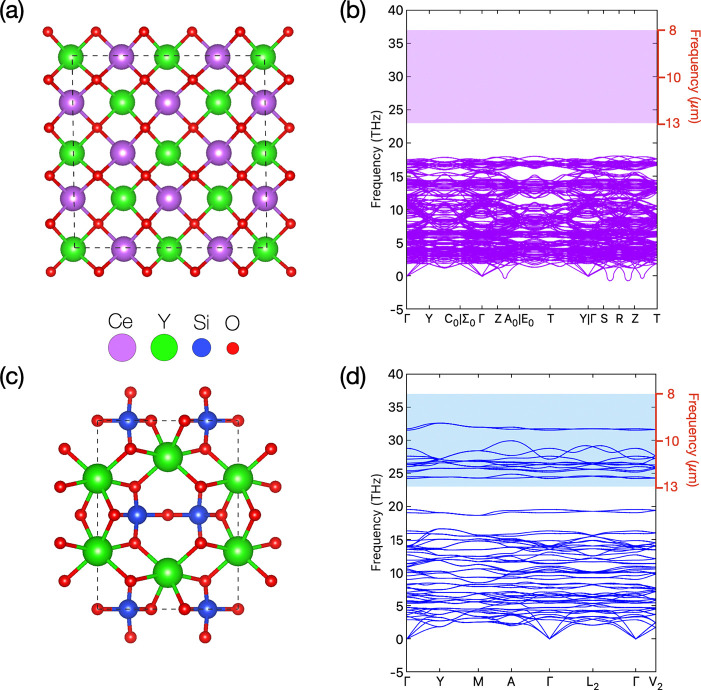
(a) PAW/PBE optimized structure of fluorite Y_2_Ce_2_O_7_ along with its (b) phonon band structure. (The wavelength corresponding to phonon frequency is highlighted with the red right-*y* axis, and the sky window ranging in 8–13 μm is depicted by the shaded region with light purple color.) (c) PAW/PBE optimized structure of monoclinic, thortveitite-like, Y_2_Si_2_O_7_ along with its (d) phonon band structure. (The sky window ranging in 8–13 μm is depicted by shaded region with cyan color.) [See Table 1S for the high-symmetry k-point coordinates of the Brillouin zones.]

Building up the appropriate atomic structure is the first step toward reliable prediction of the thermodynamic, kinetic, electronic, and phonon properties of HEOs. Accordingly, moving from available experimental data^43^ we initially calculated the structural parameters of fluorite Y_2_Ce_2_O_7_ polymorph (space group *Fm*3*m*, 227), obtaining results (*a* = 10.88 Å, @PAW/PBE) in agreement with previous^61,62^ and with our experimental XRD data (see Figure 1). The optimized fluorite Y_2_Ce_2_O_7_ structure is reported in Figure 2a. The HSE06 (PAW/PBE) calculated bandgap (direct at Γ) is 3.28 eV (1.54 eV) perfectly matching the experimental gap (3.29 eV).^43^

The phonon dispersion (Figure 2b) along the high-symmetry path in the first BZ shows the absence of vibrational modes in the 8–13 μm range, indicating a lack of IR-active modes in the sky–window region. This result very interestingly reveals that the presence of such modes cannot be ascribed to the pure Y_2_Ce_2_O_7_ fluorite polymorph.^43^ They are indeed likely to be due to the coexistence of other fluorite-derived polymorph (nominally, pyrochlore and “defect fluorite”)^63^ domains in the experimental samples that are difficult to resolve.^64^ The extremely complex structure—not easily accessible via a single theoretical model—along with the reported tendency of fluorite-related structures to oxygen diffuse^65,66^ are the key to interpret the experimental rise of the mentioned IR-active modes in the sky–window region;^43^ otherwise, they are not detected at the theoretical level. Thus, if on one side, Y_2_Ce_2_O_7_ is experimentally demonstrated to be a valuable starting candidate for “passive cooling skins”, on the other hand, we can further improve this feature upon modulating its electronic bandgap and phonon frequencies by replacing A- and B-site cations with others able to serve our final goal. An analysis based on the abundances of individual species should prompt us to work first on A-site cation replacement, with Y being slightly less abundant than Ce. Nevertheless, we initially opted to work on the B-site cation, replacing Ce with Si. Several aspects motivate this choice: Importantly, the most stable and abundant silicon oxide, SiO_2_ (α-quartz), has already been shown to have high normal emittance in the sky–window region.^16^ Silicon and cerium have the same oxidation state (IV) making the replacement of Ce with Si a natural choice. Second, still in terms of abundances, Ce is quantitatively less abundant than Si, which is not ideal for eventual scaling up and mass production. Consequently, such B-site cation replacement represents the first step for tailoring novel materials that further improve the ability of the selective IR-emitter by populating the atmospheric sky–window frequency region.^28^ Structurally, moving from Ce to Si while keeping the stoichiometry of the initial compound is accompanied by a phase change from fluorite to the more stable monoclinic phase. Neither experimental synthetic procedures nor theoretical calculations were indeed able to detect the pristine fluorite structure also for Y_2_Si_2_O_7_; on the other hand, as stated, among all the possible structures compatible with Y_2_Si_2_O_7_ stoichiometry, our calculations reveal the monoclinic (m-) as the most stable (optimized structure reported in Figure 2c), with the stability confirmed both by the phonon band structure (reported in Figure 2d) and by the convex hull analysis reported in Table 1. It is also worth mentioning that the Y_2_Si_2_O_7_ monoclinic structure (also known as β-Y_2_Si_2_O_7_)—isotypic to thortveite Sc_2_Si_2_O_7_^67^—is well-known from previous experimental characterizations of Becerro et al.^68,69^

**Table 1:** PAW/PBE Calculated Lattice Parameters [Å], β Angle (Degrees), Energy above the Hull [eV/atoms], and Electronic Bandgap (Both PAW/PBE and HSE06, eV; *i* and *d* Refer to the *Indirect/Direct* Nature of the Bandgap) for Binary A_2_Si_2_O_7_ (A = Al, Ga, In, Sc, Y, La) Parental Compounds and Equimolar Quinary (YSGI)_2_Si_2_O_7_ HEO

**lattice parameters**	**Al**	**Ga**	**In**	**Sc**	**Y**	**La**	**Y**_0.25_**Sc**_0.25_**Ga**_0.25_**In**_0.25_
*a*	5.99	6.32	6.71	6.55	6.91	7.28	6.65
*b*	8.09	8.21	8.75	8.59	9.05	9.55	8.60
*c*	4.67	4.71	4.76	4.73	4.78	4.82	4.74
β	103.21	104.65	102.91	102.88	101.96	101.10	103.24
***E* above hull**	0.092	0.110	0.041	0.015	0.000	0.048	0.054
***E***_gap_**(PBE)**	5.21 *d*	1.40 *d*	2.68 *d*	4.29 *d*	4.86 *d*	4.62 *i*	3.55 *i*
						4.66 *d*	3.58 *d*
***E***_gap_**(HSE06)**	7.04 *d*	5.29 *d*	4.47 *d*	6.03 *d*	6.54 *d*	6.27 *i*	5.36 *i*
						6.31 *d*	5.37 *d*

A direct comparison in terms of phonon dispersion between m-Y_2_Si_2_O_7_ and the cubic Y_2_Ce_2_O_7_ in the 8–13 μm region clearly shows a marked abundance of optical modes for the former compound, thus indicating absorption of a broader wavelength range of mid-infrared (MIR) phonons in m-Y_2_Si_2_O_7_. The initial B-site substitution, if on one side has the effect of reducing the symmetry of the final compound from cubic to monoclinic, on the other hand at the same time has the important consequence, fundamental for our analysis, of dragging the phonon frequencies into the long-waves region even if their distribution and density are still rather poor. Therefore, to fix such an issue, we may think of proceeding with subsequent partial substitutions on the A-site in order to achieve a better coverage of the shaded sky window region responsible for the MIR activity of such compounds. Since monoclinic Y_2_Si_2_O_7_ is isotypic to thortveitite Sc_2_Si_2_O_7_,^67^ we can operate similar substitutions of the rare-earth A-site metal using other +3 oxidation state ions, i.e., Al, La, Sc, Ga, and In. In Table 1 we report calculated bandgaps and optimized lattice parameters for cubic Al_2_Si_2_O_7_, Ga_2_Si_2_O_7_, In_2_Si_2_O_7_, Sc_2_Si_2_O_7_, Y_2_Si_2_O_7_, and La_2_Si_2_O_7_, respectively, while for the same systems, Figure S1 in the Supporting Information shows phonon frequencies.

Positive combining effects can be achieved upon binary mixing on the @A-site (thereby providing a final ternary compound), as shown in Figure S2. Indeed, such operation on the @A-site (as well as the simple substitution) clearly affects the vibrational frequencies and their distribution. Anyway, the two main requirements of an efficient radiator are not met simultaneously: we obtain either wide band gap compounds (see Table S2 in the Supporting Information) or long-wave phonon frequencies homogeneously spread over the 8–13 μm range.

Therefore, it is necessary to move from m-Y_2_Si_2_O_7_ to an entropy-stabilized oxide that embodies all of the features of a good MIR radiator and that can be formed when several constituting binary oxides (generally five or more) are mixed in near equimolar amounts and heated at high temperature (nonequimolar substitutions, such as ternary mixing on the @A-site, which provides a quaternary final compound, are beyond the scope of this work and will not be treated). To this aim, we exploited SQS quinary HEO random solid solution with quaternary mixing on the @A-site to ensure equimolarity (A = Ga, In, Sc, Y) using 22 atom unit cells for generating the monoclinic lattice. The relaxed lattice vectors of the final optimized structure of quinary (YSGI)_2_Si_2_O_7_ SQS model HEO in its monoclinic phase are listed in Table 1.

**Figure 3:**
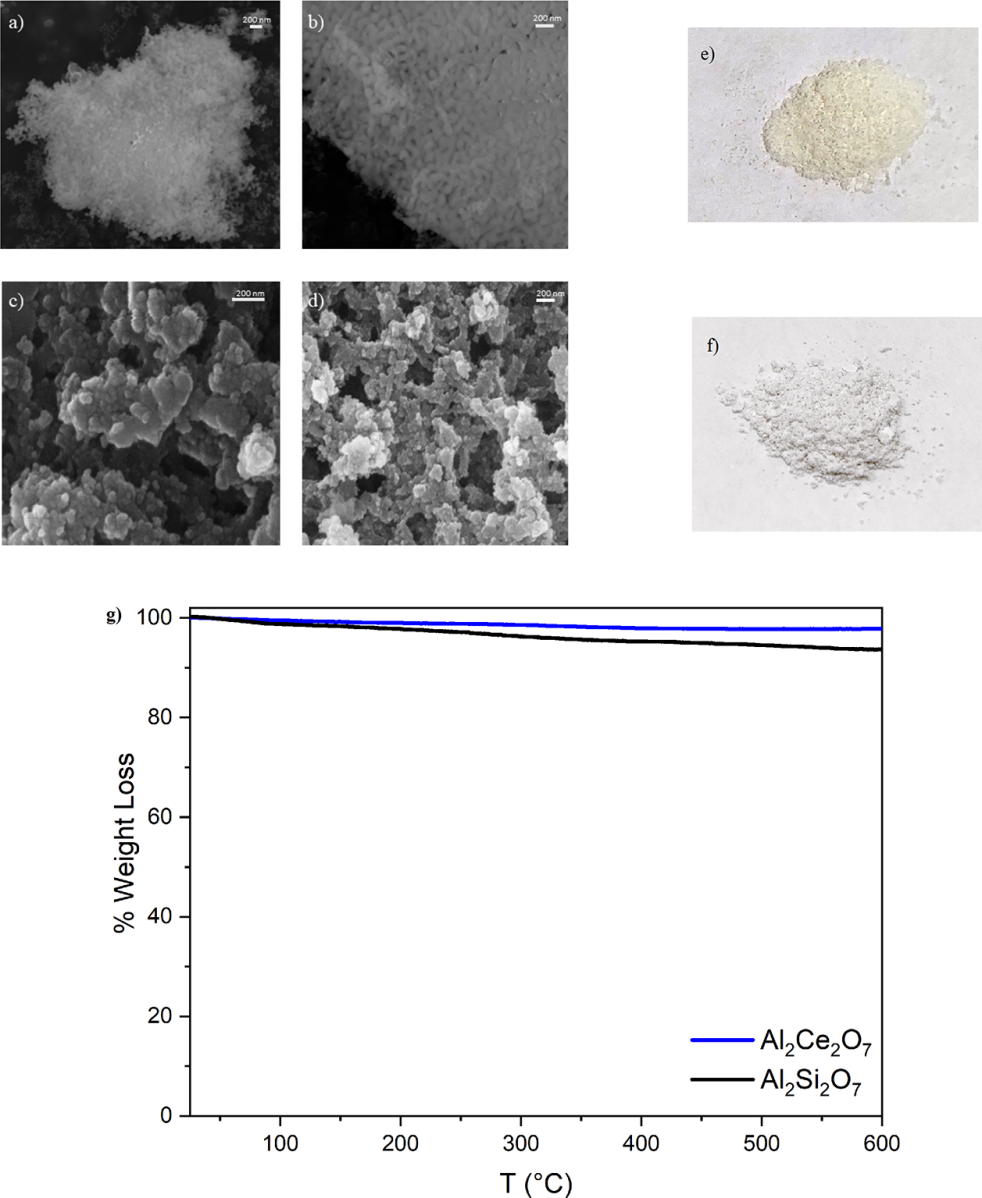
SEM images of (a), (b) Al_2_Ce_2_O_7_ and (c), (d) Al_2_Si_2_O_7_. Powders of (e) Al_2_Si_2_O_7_ and (f) Al_2_Ce_2_O_7_. (g) TGA curves of Al_2_Ce_2_O_7_ (blue) and Al_2_Si_2_O_7_ (black), respectively.

The thermodynamic stability of an alloy is fundamental as it may influence its life cycle as well as the possibility of synthesizing the compound itself. This can be measured by the driving force for a HEO to dissociate into its most stable combination of parental compounds. In practice, this is determined by comparing the energy of the HEO with the convex energy hull of all of the constituent compounds in the relative phase diagram. Table 1 provides the energy above the hull of parental compounds and the corresponding final HEO. The ground state hulls were determined from all the calculated compounds in the Materials Project database.^60^ Small energies above the hull imply that the specific material has an enhanced chance of being stabilized. When A = Al, Ga, the structures are more thermodynamically unstable (0.092 and 0.110 eV/atoms, respectively) compared to the other equivalent compounds (A = In, Sc, Y, and La), suggesting the difficulty to synthesize the former structures (A = Al, Ga). Preliminary experimental data based on our theoretical analysis show interesting results also for A = Al binary compounds: both Al_2_Ce_2_O_7_ and Al_2_Si_2_O_7_ have been indeed synthesized and partially characterized, as shown in Figure 3 and Figures S3–S5 (in the Supporting Information the experimental procedure is reported).

Scanning electron microscopy (SEM) measurements of Al_2_Ce_2_O_7_ (Figures 3a,b) and Al_2_Si_2_O_7_ (Figures 3c,d) indicate the uniformity of the particles with a semispherical shape and size of ≈30 nm. The different colors between Al_2_Ce_2_O_7_ (pale yellow, Figure 3e) and Al_2_Si_2_O_7_ (white, Figure 3f) are tentatively ascribed to a different structural fashion: a layered structure is likely to characterize Al_2_Ce_2_O_7_.

Additionally, a straightforward structural attribution to Al_2_Si_2_O_7_ is more uncertain and cumbersome (see the XRD analysis in Figure 4S in the Supporting Information). Such uncertainty is clearly due to the formation of sillimanite (Al_2_O_3_·SiO_2_) domains and of other side products that may significantly impact the final attribution. Attempts to improve the selectivity toward the thortveitite structure—both increasing the number of washing cycles to eliminate almost completely the residues and, alternatively, reaching higher temperatures of calcination—are nowadays in progress.

Finally, the thermogravimetric analysis (Figure 3g) shows the thermal stability of the compounds over a broad range of temperatures, up to 600 °C.

IR radiation (i.e., “thermal radiation”) release is ascribed to atomic oscillations/vibrations. Experimentally, the dominant spectral range of the sky window is between 8 and 13 μm, where phonon (or vibrational) resonance peaks are essential for passive radiative cooling. At those peaks, photons are absorbed while interacting with phonons or vibrons, leading to a high absorptivity and emissivity. It is thus straightforward that the relationship between the structure of our binary compounds and their phonon spectra is the key feature for fine-tuning more complex HEOs.

**Figure 4:**
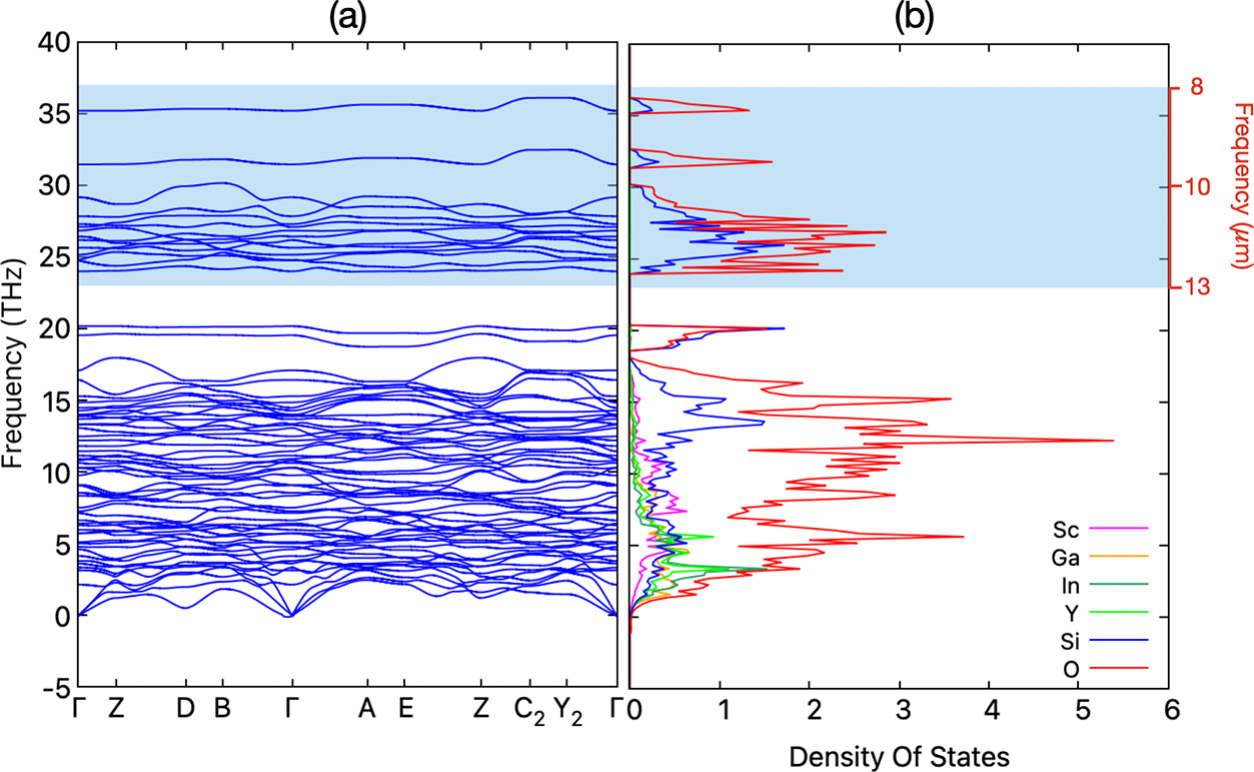
(a) Phonon band structure for (Y_0.25_Sc_0.25_Ga_0.25_In_0.25_)_2_Si_2_O_7_ HEO sampled along the high-symmetry k-point path (see Table 1S for the high-symmetry k-point coordinates) and (b) its projected density of states. [The sky window ranging in 8–13 μm is depicted by the shaded region with cyan color.]

**Figure 5:**
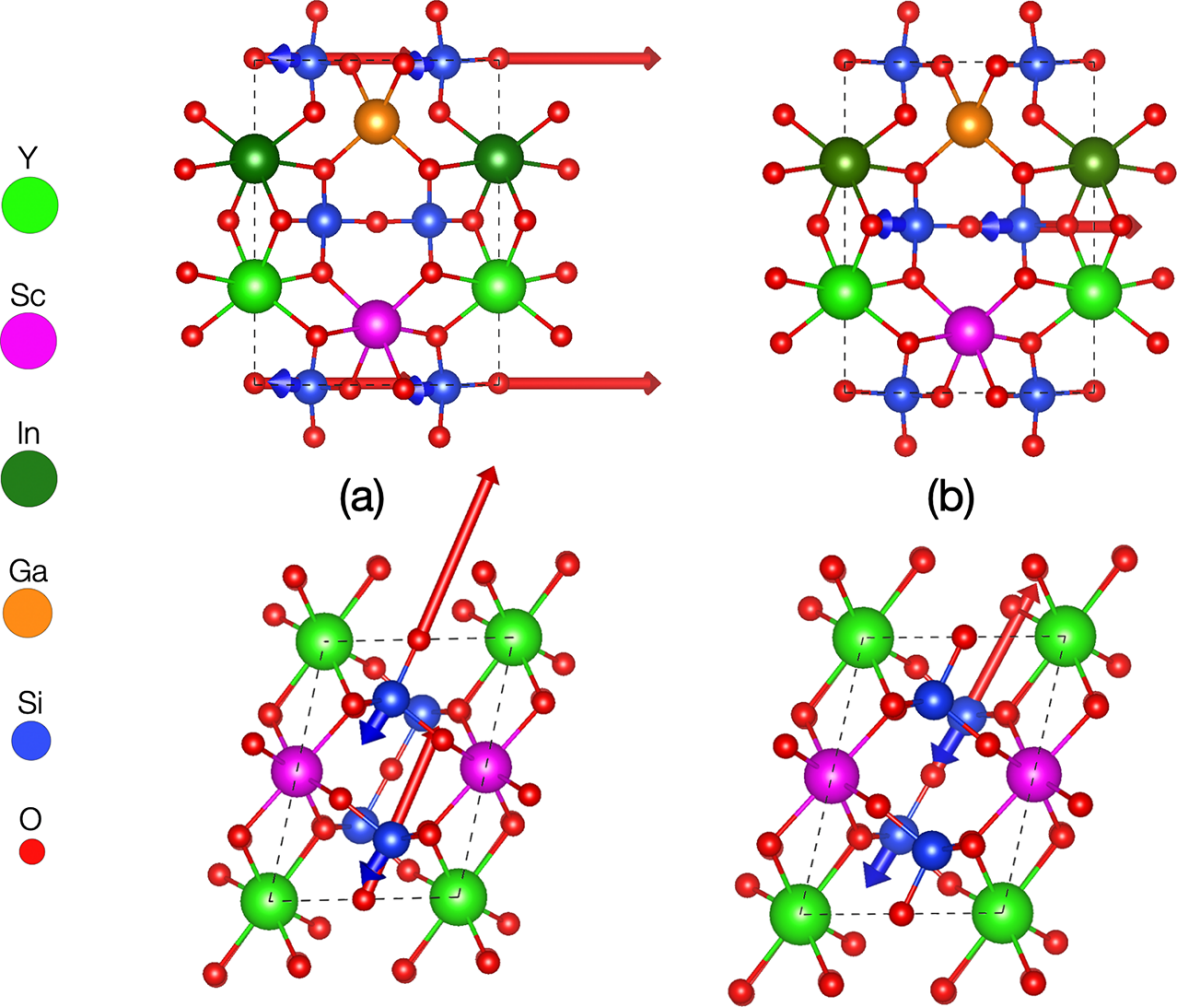
(a) Visualization of eigenvectors of phonon dispersion for (Y_0.25_Sc_0.25_Ga_0.25_In_0.25_)_2_Si_2_O_7_ HEO at **q** = 0 for ω = 35.16 THz (=8.53 μm) and (b) ω = 31.43 THz (=9.54 μm). Lateral (top) and top (bottom) orientation views for the lattice are shown.

The results of total DOS and partial DOS for (YSGI)_2_Si_2_O_7_ are reported in Figure 4 and specifically examined to better understand the origin of the peaks in the phonon density of states (DOS). As can be seen, Figure 4 depicts two distinct frequency regions. In general, the main contribution to phonon DOS in the first region (below 20 THz) stems from oxygen vibrational modes that hybridize with metallic A-site (A = Sc, Ga, Y, In) and B-site (B = Si) ones. At higher frequencies, the A-site contribution tends to vanish, being progressively replaced by Si vibrational modes. This is particularly effective in the 23–37 THz region (corresponding to the sky–window region), where the phonon DOS clearly shows contributions from silicon and oxygen only. From the phonon spectra of the parent compounds (reported in Figure S1 in the Supporting Information), one can observe that the metal element (A-site) is responsible for the shift of the higher frequency region peaks (specific phonon modes decrease from Al (atomic mass *M* = 13) to In (*M* = 49) and further from Sc (*M* = 21) to La (*M* = 57)) while the entropy ascribed to the mixing process of the HEO formation is responsible for their splitting. Moreover, according to the space-group analysis and the eigenvectors representation of the phonon dispersion for the zone-center (**q** = 0), the frequencies at ω = 31.43 THz (=9.54 μm) and at ω = 35.16 THz (=8.53 μm) correspond to optical longitudinal vibrations with B_u_ irreducible representation (see Figure 5). These vibrational modes are antisymmetric by inversion and therefore IR active. It is also worth stressing that, even though (monoclinic, *C*2/*m*) Ga_2_Si_2_O_7_ is not kinetically stable at determined ambient pressure and temperature (as shown in Figure S1 in the Supporting Information), the final HEO entails the possibility of stabilizing all the parent binary compounds into a single solid phase, as demonstrated still in Figure S1 in the Supporting Information. Indeed, this is a direct effect of the value of the configurational entropy, which becomes exceptionally large in the case of a multicomponent (generally up to 5 components) mixture, compensating for the unfavorable corresponding enthalpy contribution in the Gibbs free-energy equation.

When focusing on the electronic features of the ideal radiator, one aims to obtain materials with sufficiently wide electronic bandgaps to minimize the solar absorption in the UV–VIS region. Still, if some of the considered materials were to absorb (and consequently emit) some light for specific purposes (e.g., brightening up a room), direct-bandgap materials would be preferred since an enhanced emission is accompanied by reduced heating of the material itself. Tong et al.,^28^ focusing on BaSO_4_ as a candidate for radiative cooling, predicted ideal performances for all those materials that possess bandgaps larger than 4.13 eV, i.e., energy upper bound in the solar spectrum, and smaller than that of BaSO_4_ (7.27 eV). In this sense, our HSE06 calculated bandgap for (YSGI)_2_Si_2_O_7_ turns out to be an *almost* direct compound, where the indirect (D → Γ) bandgap is calculated to be 5.37 eV, only 0.01 eV smaller than the direct (Γ → Γ) calculated one (5.36 eV; see Table 1). Another relevant feature to discuss is the highly desirable flatness of the bandedges of the material. Effective masses of the two carriers should be ideally infinite to maximize the recombination of any eventual excitation from the VBM to the CBM. This requirement is only partially fulfilled by our HEO since the PAW/PBE calculated electronic band structure reported in Figure S6 in the Supporting Information clearly shows a VBM almost flat with a hole effective mass that is 59.06 *m*_0_, while the CBM shows a more dispersive behavior with an electron effective mass that is ∼0.650 *m*_0_. Nevertheless, a comparison with photocarrier effective masses calculated for α-quartz^70^ and orthorhombic BaSO_4_ at the same level of theory reveals faster carriers for these two materials (*m*_*h*_ = 11.5 *m*_0_; *m*_*e*_ = 0.496 *m*_0_ for SiO_2_ and *m*_*h*_ = 2.299*m*_0_; *m*_*e*_ = 0.469*m*_0_ for BaSO_4_, respectively) further endorsing the better performances also in this respect of (YSGI)_2_Si_2_O_7_.

**Figure 6:**
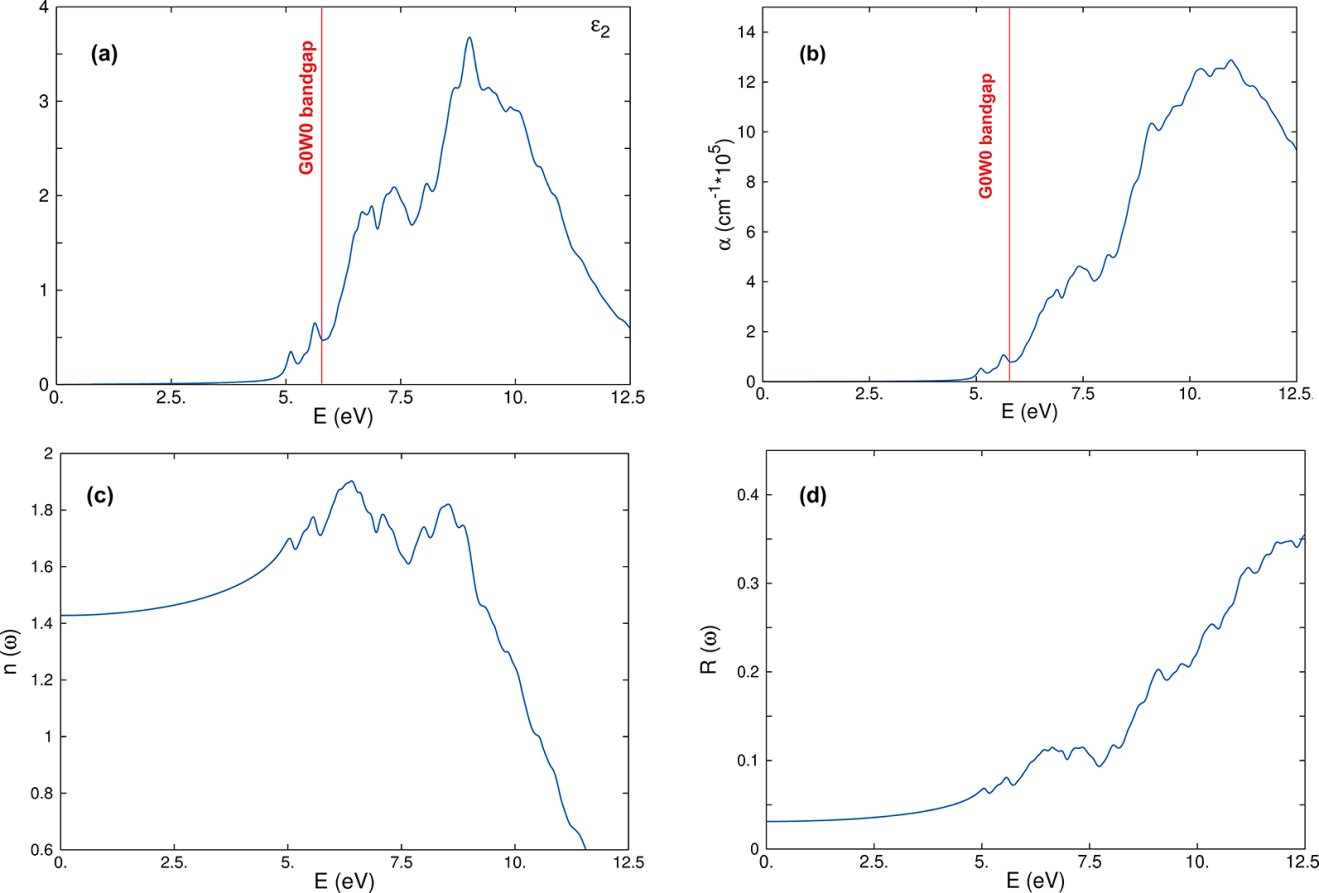
(a) Optical spectrum calculated at the BSE level for (YSGI)_2_Si_2_O_7_. The spectrum is obtained using 20 (20) occupied (unoccupied) states in the BSE matrix. (b) Absorption spectrum still calculated at the BSE level. (c) and (d) are refractive index, *n*(ω), and reflectivity, *R*(ω), respectively, obtained at the same level of theory. The red line in (a) and (b) shows the value of the QP bandgap calculated at the *G*_0_*W*_0_ level of theory.

The optical properties (imaginary part of the dielectric function, ϵ_2_, absorption, refractive index, and reflectivity) of β-(YSGI)_2_Si_2_O_7_ are reported in Figure 6. A large renormalization of the DFT bandgap is obtained when calculating the QP bandgap at the “one-shot”, *G*_0_*W*_0_, level of theory. The so-calculated bandgap (5.78 eV) is consistent with the HSE06 calculated one. For the sake of comparison, we calculated the QP bandgap for α-quartz (SiO_2_) obtaining a value of 8.76 eV, a result that well reproduces the range of the experimental values,^71,72^ validating our setup (see Figures S7–S10 in the Supporting Information).

Finally, we have calculated the Born effective charge (Z*) of the constituent ions for the β-(YSGI)_2_Si_2_O_7_ structure. The static charges or the nominal charges of @A-site (A = Y, Sc, In, Ga), @B-site (B = Si) and oxygen in (YSGI)_2_Si_2_O_7_ are +3, +4 and −2, respectively. From the values of Z* tensors reported in Table S2 in the Supporting Information, in our theoretical model, constituent ions developed a maximum effective charge of 4.10 for the @A-site, 3.69 for the @B-site, and −3.37 for the @C-site, showing +37%, −8%, and +68% change from the static charge, respectively, for each ion. Furthermore, the calculated dielectric tensors (Table S3 in the Supporting Information), show a slightly anisotropic behavior with presence of off-diagonal tensor elements, coherently with the monoclinic character of the structure. In the transparent spectral range (*E* < 4 eV), this off-diagonal element is small (∼0.04). With the knowledge of Z* values and the dielectric constant, we can apply the so-called no analytic term correction (NAC). As shown in Figure S11 in the Supporting Information, one can see that the NAC term removes the slightly imaginary strains of the acoustic modes at Γ and shows the absence of and longitudinal optical–transverse optical (LO–TO) splitting at the Γ point, which is consistent with the nonpolar character of β-(YSGI)_2_Si_2_O_7_.

## Conclusions

Passive daytime radiative cooling is a promising phenomenon recently proposed for abating urban overheating and reducing the consequent thermal comfort deterioration in the built environment. Adequate passive coolers should reflect most of the incoming UV, VIS, and near-infrared radiation, while dissipating excess heat selectively in the specific waveband range of the atmospheric window. Designing, producing, and testing such a material is a compelling challenge for researchers worldwide that can only be addressed by tackling our current lack of knowledge about candidate materials’ structural and optoelectronic features. Motivated by the impressive prospect of selective daytime radiative cooling, this work proposes high-entropy oxides as a class of potential candidates to this aim.

By combining density functional theory, many-body perturbation theory, and the finite difference method, we investigate phonon band structure, electronic bandgap, optical features, and carrier effective masses aiming to predict the expected performance of the proposed compounds. More in detail, moving from the most recent experimental results in the literature, we have characterized structurally and electronically the archetypal compound Y_2_Ce_2_O_7_. Results show that further substitutions in both A-site and B-site ionic positions lead to impressive changes in the phonon band structure, populating with IR-active frequency modes the region of interest (atmospheric sky–window). Overall, our finely tailored material (Y_0.25_Sc_0.25_Ga_0.25_In_0.25_)_2_Si_2_O_7_ satisfies the optimal electronic properties and homogeneous phonon distribution in the region of interest as requested for an efficient process. In the present work, we propose ab initio calculations as an effective tool for advanced material design and development in the field of radiative cooling. We therefore expanded the possible technological applications of high-entropy oxides, an emerging class of materials, investigating their potential for daytime passive radiative cooling applications. Finally, we explore the possible benefits of this relevant technology that could effectively mitigate detrimental urban overheating effects and reduce anthropogenic CO_2_ production.

## Supporting Information Available

Experimental procedure; FTIR spectra, solar reflection and MIR emittance; phonon band structure of parental compounds (binary and ternary oxides); Brillouin zone of the investigated compounds; bare PAW/PBE bandgaps; Born effective charges; BSE calculated optical spectrum, absorption spectrum, refractive index, and reflectivity for SiO_2_ (PDF)
