# Supplementary material for: Tailoring High-Entropy Oxides as Emerging Radiative Materials for Daytime Passive Cooling
Source: Chem Mater. 2023 Oct 23;35(24):10384–93. doi: 10.1021/acs.chemmater.3c01205 (PMC10753767; doi:10.1021/acs.chemmater.3c01205)
Supplement: Supplementary file 1 — cm3c01205_si_001.pdf [file cm3c01205_si_001.pdf]

Supporting Information: Tailoring High-Entropy Oxides as Emerging Radiative Materials for Daytime Passive Cooling

Costanza Borghesi,[†,‡] Claudia Fabiani,[¶,‡] Roberto Bondi,[§] Loredana Latterini,[§] Ivano E. Castelli,[*,∥] Anna Laura Pisello,[*,¶,‡] and Giacomo Giorgi[*,†,‡,⊥]

†*Department of Civil & Environmental Engineering (DICA), Università degli Studi di Perugia, Via G. Duranti 93, 06125 Perugia, Italy.*

‡*CIRIAF – Interuniversity Research Centre, University of Perugia, Perugia, Italy.*

¶*Department of Engineering, Università degli Studi di Perugia, Via G. Duranti 93, 06125 Perugia, Italy.*

§*Nano4Light Lab, Department of Chemistry, Biology and Biotechnology, University of Perugia, Via Elce di sotto 8, Perugia, 06123, Italy.*

∥*Department of Energy Conversion and Storage, Technical University of Denmark, DK-2800 Kgs. Lyngby, Denmark*

⊥*CNR-SCITEC, 06123 Perugia, Italy*

E-mail: ivca@dtu.dk; anna.pisello@unipg.it; giacomo.giorgi@unipg.it

Phone: +39 075 5853836

Experimental Section

Materials and Methods All the reagents are commercially available and used without further purification. All the reagents were purchased from Merck. X-ray diffraction (XRD) patterns of the samples were recorded using a Malvern PANalytical Empyrean diffractometer with BraggBrentano geometry. The diffractogram was recorded in 20-70° 2Θ range with a step size of 0.013° and an integration time of 79 s/step using a PIXcel3D-Medipix3 solid-state detector and a Cu anode. The dimensions and morphology of the compounds were measured through a FE-SEM (FEG LEO 1525, Zeiss, Oberkochen, Germany). Thermogravimetric analysis were carried out with a Netzsch STA2500 Regulus thermoanalyzer under a 5 mL min^{-1} air flux with a heating rate of 10 °C min^{-1}. For the measurements of solar reflectance a JETI Specbos 160 1211UV spectroradiometer was used, while the FTIR spectra and MIR emittance measurements were obtained with a PerkinElmer Spectrum 3™ MIR/NIR/FIR system equipped with a 3-inch diameter highly reflective gold-coated Mid-IR IntegratIR™ sphere (PIKE Technologies).

Synthesis of $Y_2Ce_2O_7$ and $Al_2Ce_2O_7$. $Y_2Ce_2O_7$ and $Al_2Ce_2O_7$ were synthesized following the sol-gel procedure described by Dang et al.[1] 0.5 mmol of $Y(NO_3)_3\cdot 6H_2O$ and 0.5 mmol of $Ce(NO_3)_3\cdot 6H_2O$ are put into 10 mL of deionized water and stirred. After 10 minutes, 1 mmol of citric acid is added to the solution. When the acid is dissolved completely, 3 g of glucose in 100 mL of deionized water and 2 mmol of acrylamide are added. Following the addition of glucose and acrylamide, the pH value of the solution is adjusted to ≈ 7 with NH_4OH. The solution is then heated at 80°C in a water bath for 3h to become a gel, and then the obtained gel is dried at 120°C in an oven overnight. Finally, the gel precursor is grinded into powder and calcined at 900°C for 4h in a muffle furnace to achieve the final product. For the synthesis of $Al_2Ce_2O_7$ the same procedure has been followed, using $Al(NO_3)_3\cdot 9H_2O$ instead of $Y(NO_3)_3\cdot 6H_2O$

Synthesis of $Al_2Si_2O_7$.$Al_2Si_2O_7$ was synthesized with a similar sol-gel method introducing some modifications. 0.5 mmol of tetraethoxysilane (TEOS) are dissolved in 10 mL

of ethanol and stirred for 24h. Then, the TEOS solution is added into a 0.05 M aqueous solution of $Al(NO_3)_3 \cdot 9H_2O$ and stirred for 20 minutes. After this, the procedure is the same as described above for the synthesis of $Y_2Ce_2O_7$.

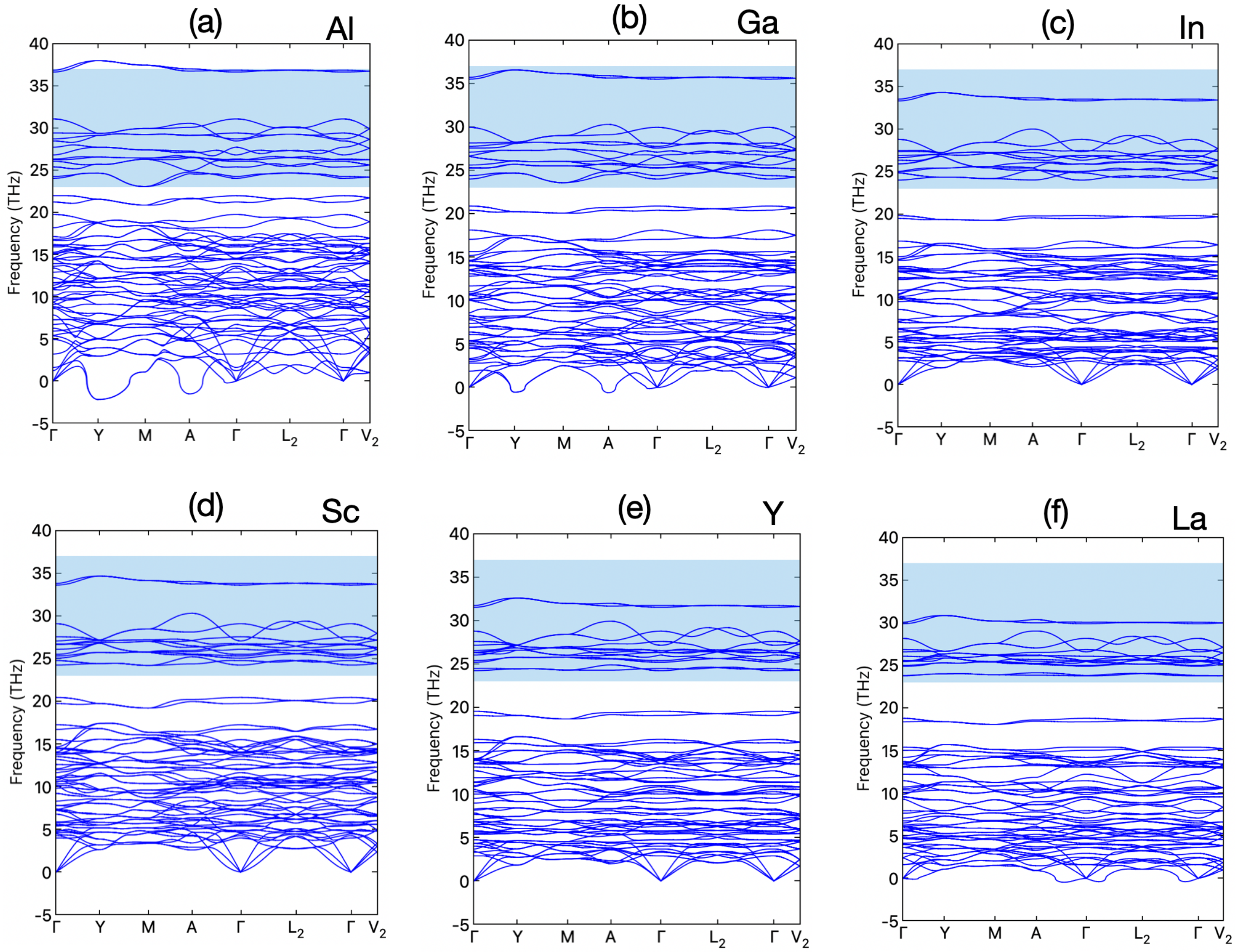

Figure S1: Phonon bandstructure for **binary** $A_2Si_2O_7$: (a) A=Al, (b) A=Ga, (c) A=In, (d) A=Sc, (e) A=Y, (f) A=La. (See Table S1 for the high symmetry k-points coordinates of the Brillouin Zone).

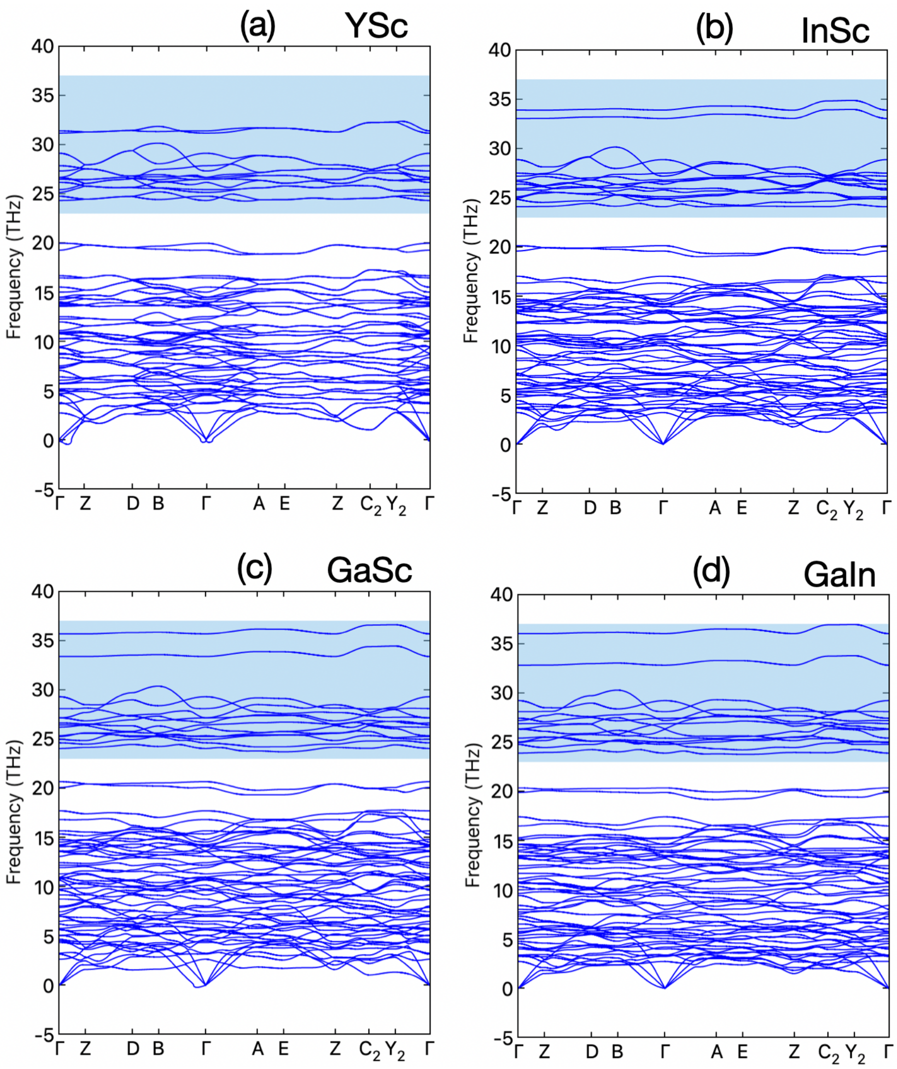

Figure S2: Phonon bandstructure for **ternary** $(A_{0.5}A'_{0.5})_2Si_2O_7$: (a) A=Y, A'=Sc, (b) A=In, A'=Sc, (c) A=Ga, A'=Sc, (d) A=Ga, A'=In. (See Table S1 for the high symmetry k-points coordinates of the Brillouin Zone).

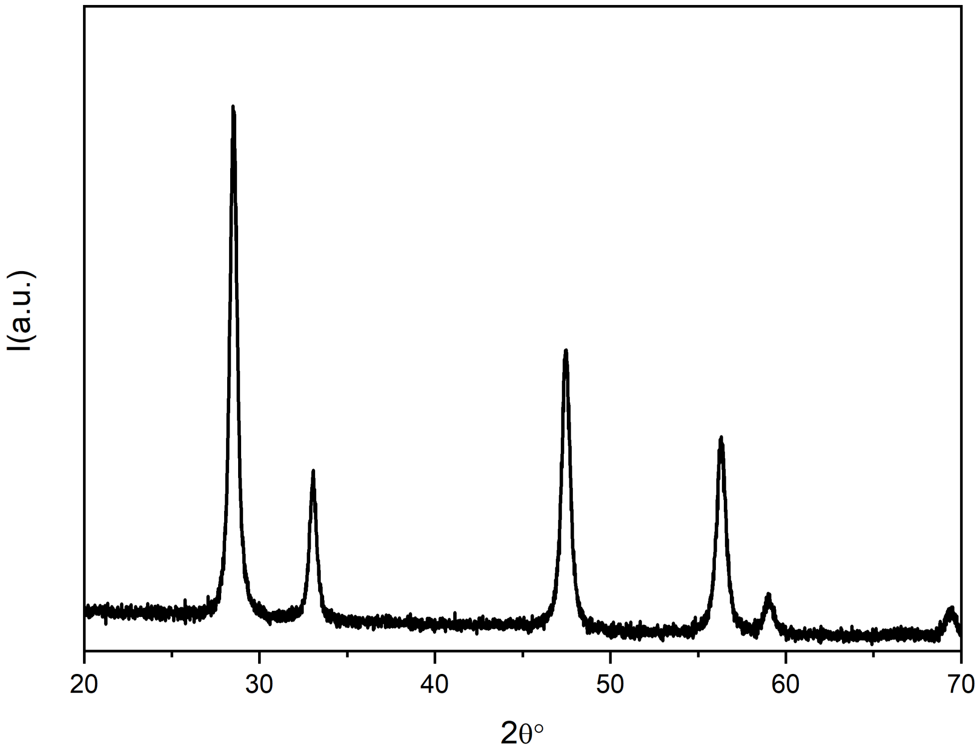

Figure S3: XRD pattern of as-synthesized $Al_2Ce_2O_7$

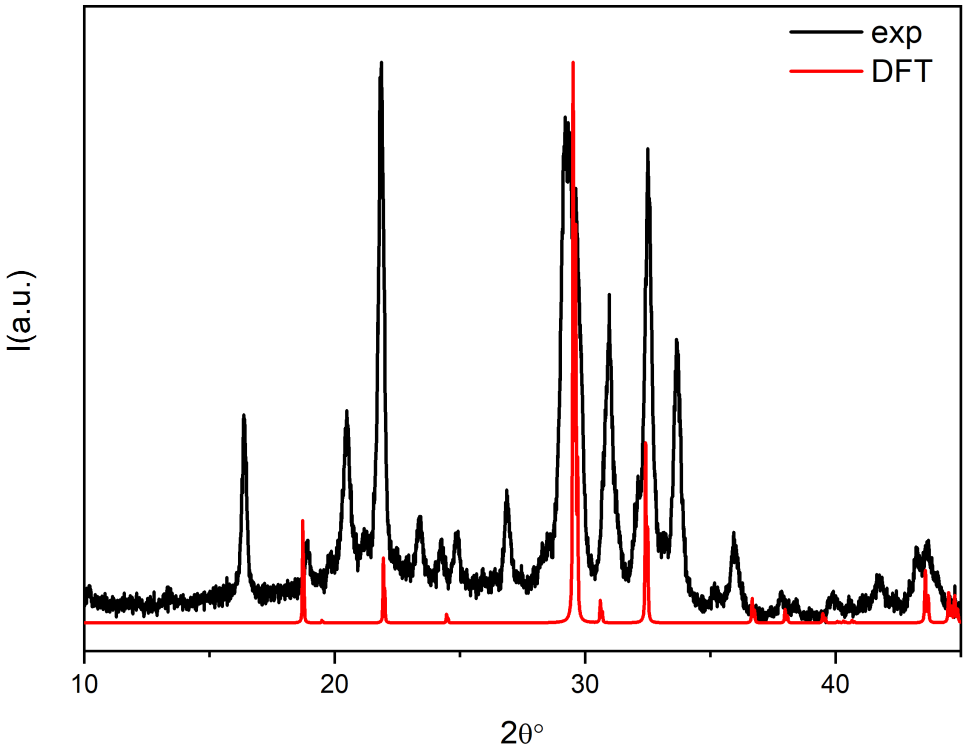

Figure S4: Comparison of XRD patterns of as-synthesized $Al_2Si_2O_7$ (black) and theoretical $Al_2Si_2O_7$ (red)

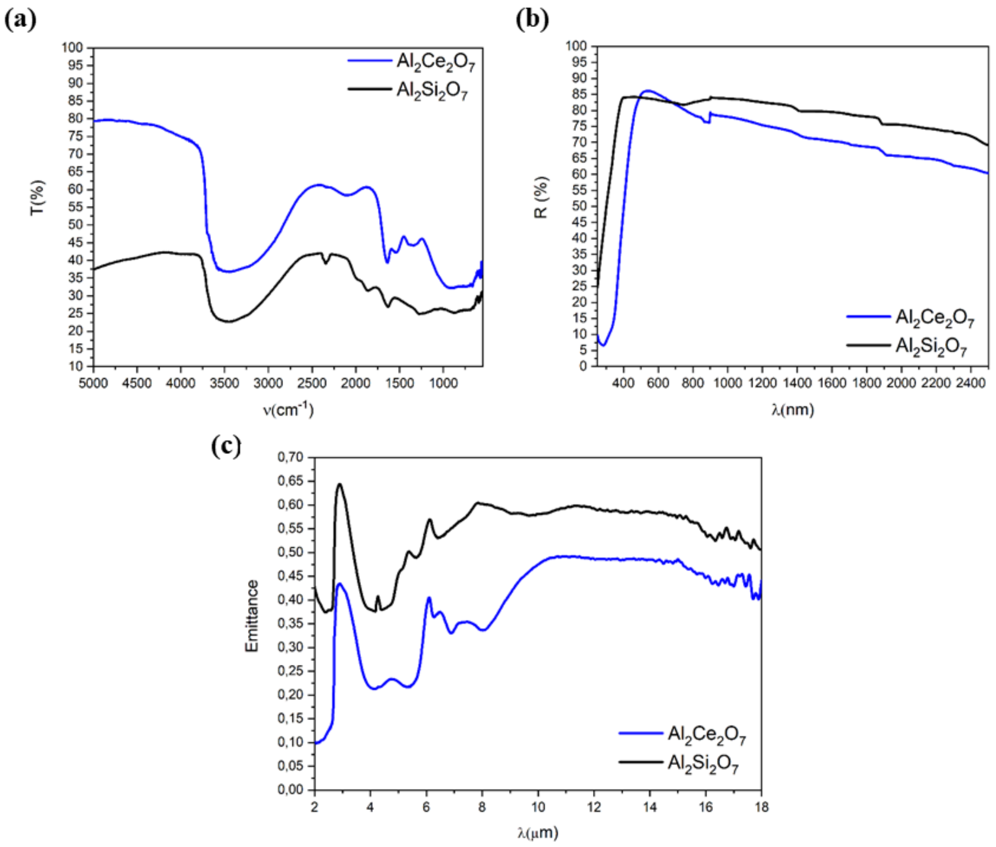

Figure S5: (a) FTIR spectra, (b) solar reflection and (c) MIR emittance of the as-synthesized samples $Al_2Si_2O_7$ (black) and $Al_2Ce_2O_7$(blue)

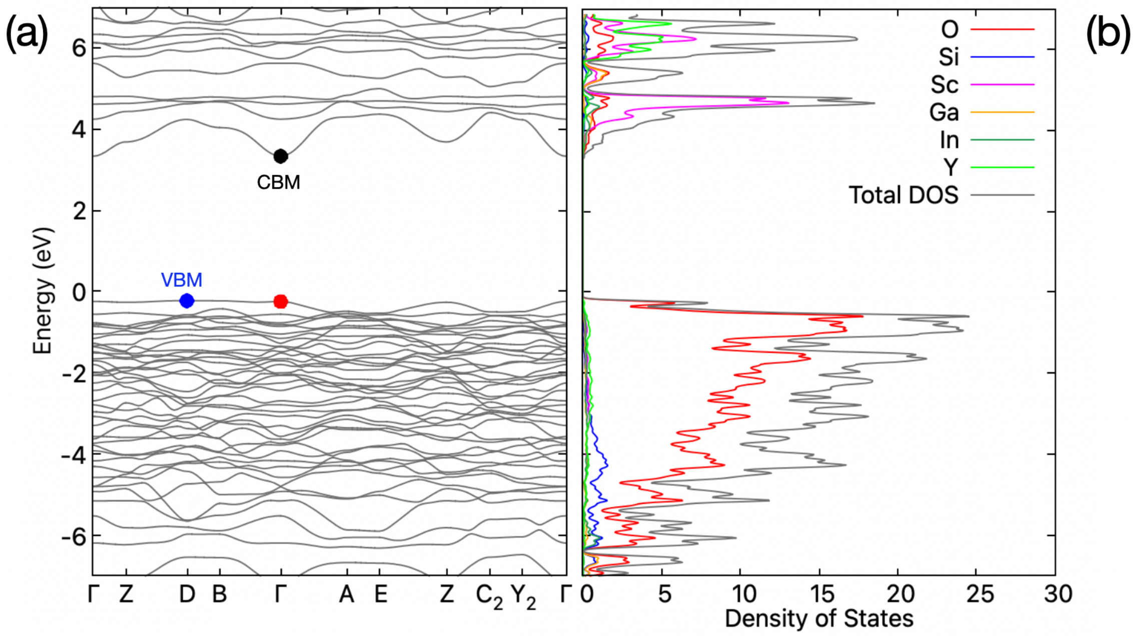

Figure S6: (a) PAW/PBE calculated electronic bandstructure for $(Y_{0.25}Sc_{0.25}Ga_{0.25}In_{0.25})_2Si_2O_7$ HEO and (b) its projected density of states. CBM (at Γ) and VBM (both at Γ and at D point (0.0 0.5 0.5)) are indicated with colored dots.

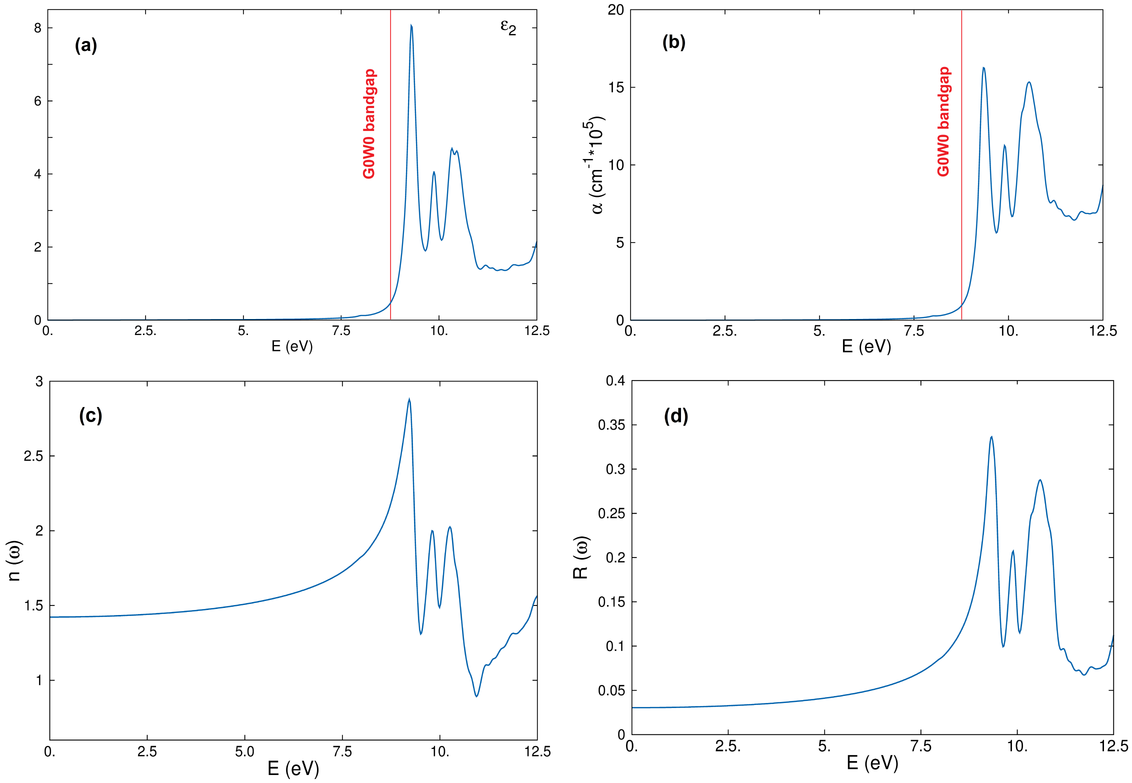

Figure S7: (a): Optical spectrum calculated at the BSE level for SiO_2; (b) Absorption spectrum still calculated at the BSE level; (c) and (d) are refractive index, n(ω) and reflectivity, R(ω), respectively, obtained at the same level of theory.The red line in (a) and (b) shows the value of the QP bandgap calculated at the G_0W_0 level of theory. Spectra are calculated using 15 (15) occupied (unoccupied) states in the BSE matrix. 24 k-points were used to sample the Brillouin Zone. 960 Bands were included in the calculations. All the remaining parameters are the same as reported in Computational Details section.

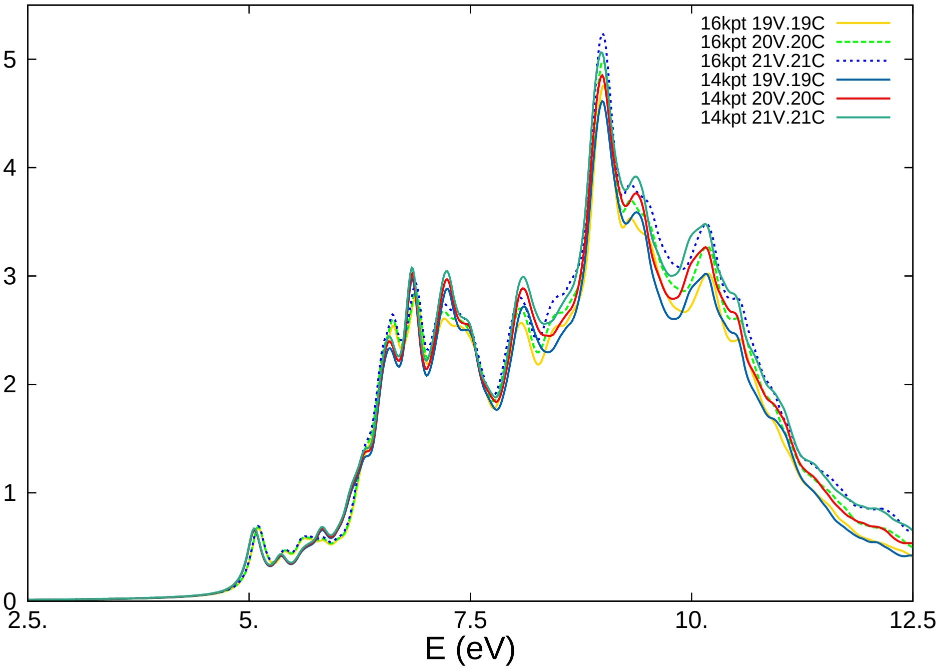

Figure S8: Convergence test for $(Y_{0.25}Sc_{0.25}Ga_{0.25}In_{0.25})_2Si_2O_7$. Here, the xx component of the imaginary part of the dielectric function, ϵ_2, is calculated by solving the BSE as function of the number of k-points (n kpt) used to sample the Brillouin Zone and of the number of occupied (n V) and empty (n C) states similarly exploited for the same calculation.

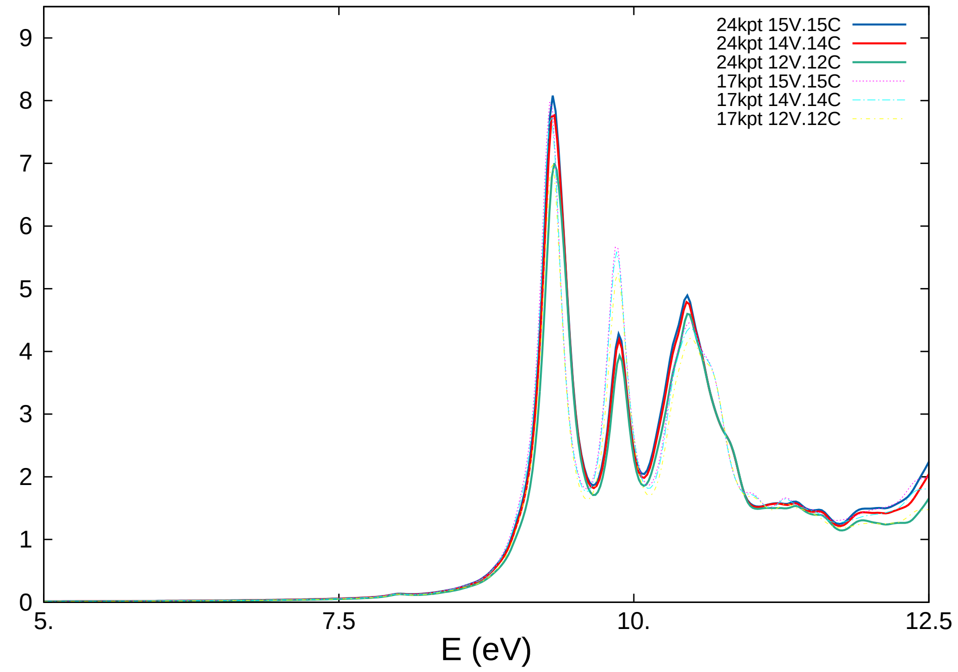

Figure S9: Same analysis of Figure S8 but for the case of SiO_2 (α-quartz).

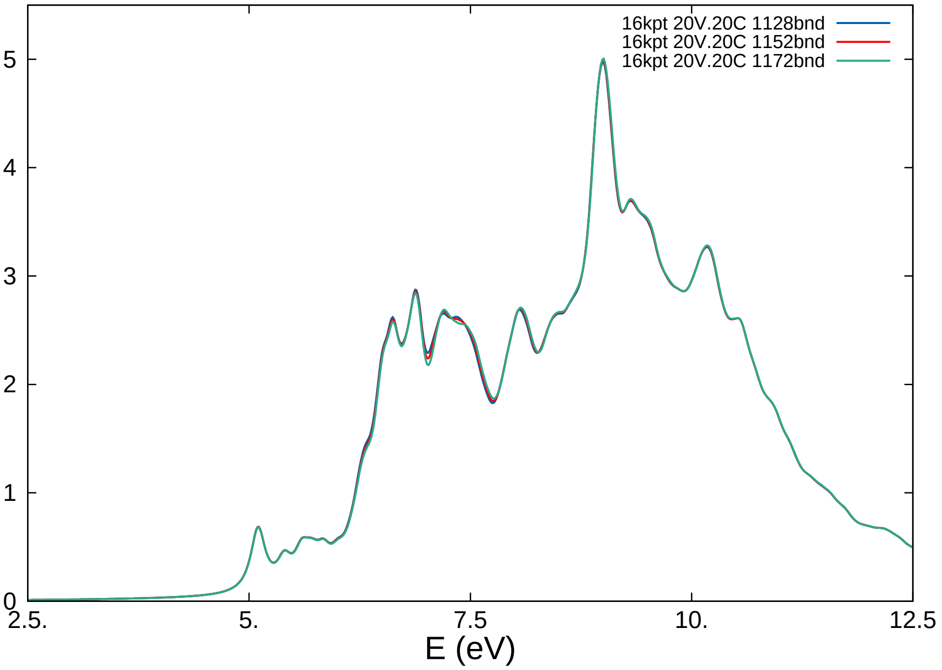

Figure S10: Convergence test for $(Y_{0.25}Sc_{0.25}Ga_{0.25}In_{0.25})_2Si_2O_7$. in terms of number of bands employed to calculate the imaginary part of the dielectric function, ϵ_2. The plot reports the xx component calculated at the BSE level of theory.

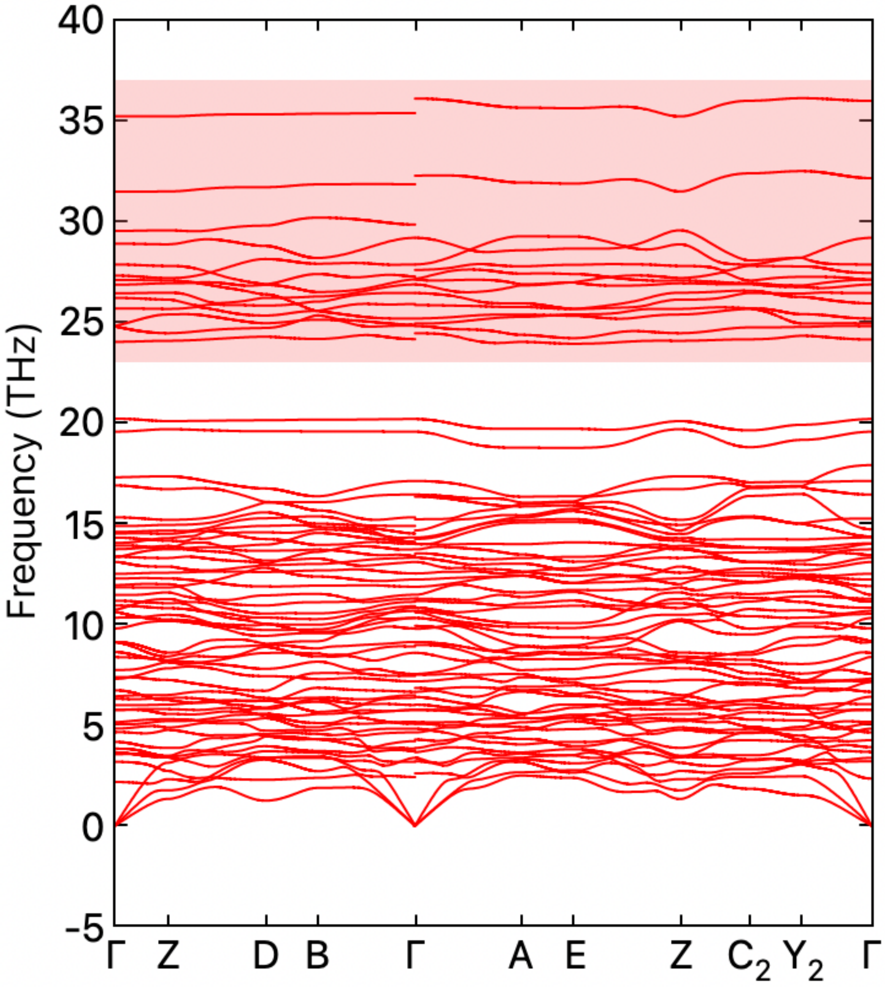

Figure S11: Phonon bandstructure with NAC term correction for $(Y_{0.25}Sc_{0.25}Ga_{0.25}In_{0.25})_2Si_2O_7$. (See Table S1 for the high symmetry k-points coordinates of the Brillouin Zone).

Table S1: High-symmetry k-points path for the Brillouin Zone sampling for $Y_2Ce_2O_7$, for the parent compounds $A_2Si_2O_7$ (A=Al, Ga, In, Sc, Y, La), and for the final HEO $(YSGI)_2Si_2O_7$.

Structure				
$\mathbf{Y_2Ce_2O_7}$	Γ	0.0	0.0	0.0
	**X**	0.5	0.5	-0.5
	$\mathbf{F}_2$	0.375	0.625	-0.375
	Σ_0	-0.375	0.375	0.375
	$\mathbf{Y}_0$	0.375	-0.375	0.375
	$\mathbf{U}_0$	0.625	0.375	-0.375
	**R**	0.0	0.5	0.0
	**W**	0.25	0.25	0.25
	**S**	0.5	0.0	0.0
	**T**	0.0	0.0	0.5
$\mathbf{A_2Si_2O_7}$	Γ	0.0	0.0	0.0
	**Y**	0.5	0.5	0.0
	**M**	0.5	0.5	0.5
	**A**	0.0	0.0	0.5
	$\mathbf{L}_2$	0.0	0.5	0.5
	$\mathbf{V}_2$	0.0	0.5	0.0
$\mathbf{(A_{0.5}A'_{0.5})_2Si_2O_7}$ and $\mathbf{(YSGI)_2Si_2O_7}$	Γ	0.0	0.0	0.0
	**Z**	0.0	0.5	0.0
	**D**	0.0	0.5	0.5
	**B**	0.0	0.0	0.5
	**A**	-0.5	0.0	0.5
	**E**	-0.5	0.5	0.5

Table S2: Calculated bandgaps (eV) at the PAW/PBE level for the ternary compounds.

Structure	Bandgap (eV)
$\mathbf{(Y_{0.5}Sc_{0.5})_2Si_2O_7}$	3.55
$\mathbf{(In_{0.5}Sc_{0.5})_2Si_2O_7}$	0.84
$\mathbf{(Ga_{0.5}Sc_{0.5})_2Si_2O_7}$	0.10
$\mathbf{(Ga_{0.5}In_{0.5})_2Si_2O_7}$	2.82

Table S3: Convergence of Valence Band Maximum (VBM) and Conduction Band Minimum (CBM) Energy (eV) as function of the number of k-points (NKPTS) in the Brillouin Zone in "one-shot" G_0W_0 QP calculations.

	NKPTS	VBM	CBM	Bandgap
SiO_2	27	-1.33	7.43	8.76
	24	-1.33	7.44	8.77
	17	-1.34	7.43	8.77
$(YSGI)_2Si_2O_7$	22	1.73	7.51	5.78
	16	1.73	7.52	5.79
	14	1.73	7.52	5.79
	10	1.73	7.52	5.79

Table S4: Born Effective Charge tensors (BECs) in β-$(YSGI)_2Si_2O_7$.

Ion	Z_{xx}	Z_{xy}	Z_{xz}	Z_{yx}	Z_{yy}	Z_{yz}	Z_{zx}	Z_{zy}	Z_{zz}
Sc	3.97	0.00	0.01	0.00	4.01	0.00	0.12	0.00	3.30
Ga	3.64	0.00	-0.07	0.00	3.54	0.00	0.07	0.00	2.97
In	3.59	0.00	0.03	0.00	3.39	0.00	0.10	0.00	2.98
Y	4.10	0.00	0.18	0.00	3.77	0.00	0.19	0.00	3.30
Si	3.55	0.11	-0.02	-0.02	3.22	0.05	-0.21	0.01	3.69
Si	3.55	-0.11	-0.02	0.02	3.22	-0.048	-0.21	-0.01	3.69
Si	3.58	0.02	0.02	0.00	3.41	0.06	-0.10	-0.03	3.69
Si	3.58	-0.02	0.02	-0.00	3.41	-0.06	-0.10	0.03	3.69
O	-1.85	0.10	0.11	0.12	-2.10	0.64	0.20	0.62	-1.75
O	-1.85	-0.10	0.11	-0.12	-2.10	-0.64	0.20	-0.62	-1.75
O	-2.20	0.07	0.21	0.17	-2.10	0.71	0.21	0.71	-1.88
O	-2.20	-0.07	0.21	-0.17	-2.10	-0.71	0.21	-0.71	-1.88
O	-2.163	0.25	0.06	0.33	-2.37	0.81	0.03	0.78	-1.82
O	-2.16	-0.25	0.06	-0.33	-2.37	-0.81	0.03	-0.78	-1.82
O	-1.86	0.14	0.03	0.18	-2.23	0.65	0.09	0.65	-1.71
O	-1.86	0.14	0.03	0.17	-2.23	0.65	0.09	0.65	-1.71
O	-3.37	0.00	0.59	0.00	-1.21	0.00	0.57	0.00	-1.47
O	-3.18	0.00	0.65	0.00	-1.20	0.00	0.63	0.00	-1.48
O	-1.67	0.08	-0.54	0.02	-2.03	0.11	-0.52	0.10	-2.52
O	-1.67	-0.087	-0.547	-0.02	-2.03	-0.11	-0.52	-0.10	-2.52
O	-1.75	-0.09	-0.57	-0.01	-1.95	-0.07	-0.54	-0.13	-2.51
O	-1.75	0.09	-0.57	0.01	-1.95	0.07	-0.54	0.13	-2.51

Table S5: Parameters for macroscopic static dielectric tensor of β-$(YSGI)_2Si_2O_7$.

ϵ_{xx}	ϵ_{xz}	ϵ_{yy}	ϵ_{zx}	ϵ_{zz}
3.59	0.04	3.46	0.04	3.29

References

(1) Dang, S.; Xiang, J.; Yao, H.; Yang, F.; Ye, H. Color-preserving daytime passive radiative cooling based on Fe3+-doped $Y_2Ce_2O_7$. *Energy Build.* **2022**, *259*, 111861.
